# Supplementary material for: MetaRibo-Seq measures translation in microbiomes
Source: Nat Commun. 2020 Jun 29;11:3268. doi: 10.1038/s41467-020-17081-z (PMC7324362; doi:10.1038/s41467-020-17081-z)
Supplement: Supplementary file 10 — Supplementary Data 7 [file 41467_2020_17081_MOESM10_ESM.zip › File2/Confidence_High_Taxonomy/342322_out.krona.html]

Javascript must be enabled to view this page.

members
magnitude
magnitudeUnassigned
count
unassigned
taxon
rank

342322\_out

3

2
superkingdom
3

2
phylum
1239

class
2
186801

186802
2
order

172733
1
species

SRS146764\_contig\_number\_29329

family
1
31979

1
genus
1485

1776383
species

SRS104311\_contig\_number\_contig-100\_3399.253502
1

phylum
1
201174

1760
class
1

order
1
85006

family
1
85023

genus
1
447237

species

SRS148424\_contig\_number\_contig-100\_7924.7924
1
1795630
